# New Appliances and Things Medical

**Published:** 1899-06-10

**Authors:** 


### NEW APPLIANCES AND THINGS MEDICAL.

[We shall be glad to receive, at our Office, 28 & 29, Southampton Street, Strand, London, W.C., from the manufacturers, specimens of all new preparations and appliances which may be brought out from time to time.]

### "ALLENBURYS" TOILET SOAP.

(Allen and Hanburys, Limited, Plough Court, Lombard Street, London, E.C.)

The "Allenburys" toilet soap is a good example of a high-class toilet soap. It has a pale yellow tint, which is natural to the soap, and not produced by deleterious colouring matter, and is delicately perfumed. Examination of the soap bears out the statement that it is free from all adulterations. It contained only a very small proportion of water, namely, 9·2 per cent., and was almost neutral in its reaction. The soap lathers freely, and being slightly superfatted it leaves a soft, delicate feeling on the hands, and prevents any tendency to roughness and redness of the skin after washing.

### PANOPEPTON, PEPTOGENIC POWDER, AND PEPSENSIA.

(Fairchild Brothers and Foster, New York, U.S.A. London Agents: Burroughs, Wellcome, and Co., Snow Hill, London, E.C.)

Panopepton is a completely digested food prepared from lean beef and wheat flour which have been subjected to the action of ferments. By the process adopted the foods are rendered completely soluble, and capable of being absorbed by the system without any further digestion in the stomach or intestines. Containing as it does the digested products of both proteids and carbohydrates, it may be regarded as a complete food, except for the absence of fats. Hence, for infants or grown persons whose powers of digestion are enfeebled or temporarily in abeyance, panopepton is a food of the greatest utility. Being in liquid form and not unpleasant to the taste it is readily taken. The peptogenic powder is a convenient means of bringing ordinary cow's milk to the same constituency as human milk. The process is simple. A measured quantity of the powder is added to milk properly diluted, and brought to the boil for ten minutes. By this means the deficiency of milk sugar is made good and the casein partially digested; the deficiency of cream is corrected by the addition of a further quantity. The resultant mixture very closely approximates to the proportions of human milk. The partial digestion of the casein, or clot, is often of great advantage to the infant, especially in those cases where without such artificial aid the coagula leaves the body in small undigested masses. It must be remembered, however, that the growing infant requires a modification of the proportions of proteid, sugar, and fat during its various stages, and hence slight alterations in the proportions as stated on the bottle must be made from time to time under the direction of a competent adviser. With regard to pepsensia, it may be mentioned that it is an artificial adjuvant to digestion, intended mainly to be administered by the mouth, although it may also be conveniently used for making junket or curds and whey. In addition to containing the ferments of the stomach it is a valuable stimulant, and hence is of value when taken after meals in compensating for any natural deficiency in the amount or efficiency of the natural gastric secretion. It is also highly recommended as a medium for administering iodides, bromides, and other drugs which disturb the stomach.

### EUCASIN, A NEW MILK FOOD.

(Anglo-Continental Chemical Works, Limited, 1 and 2, Rangoon Street, London, E.C.)

Eucasin is a special preparation made from milk. It is sold in tins of various sizes, and may be used and prepared in a variety of ways. It is highly digestible and nutritious, and is specially indicated in those conditions in which a milk diet is recommended, as in gout and rheumatism. For typhoid and other wasting diseases with impairment of the digestive functions Eucasin is equally valuable. As a basis for biscuits, chocolate, and cocoa, Eucasin may also be usefully employed. Being a highly concentrated food, and free from starch, it is a physiologically economical food, rapidly absorbed, and little liable to vicious formation in the digestive tract. Whether in the form of biscuits or chocolate, it should prove of value to cyclists on the road, since with a minimum of bulk they will find a maximum of nutritive value in a highly digestible form.

